# The Effect of Austempering Temperature on the Matrix Morphology and Thermal Shock Resistance of Compacted Graphite Cast Iron

**DOI:** 10.3390/ma18102200

**Published:** 2025-05-10

**Authors:** Aneta Jakubus, Marek Sławomir Soiński, Grzegorz Stradomski, Maciej Nadolski, Marek Mróz

**Affiliations:** 1 Faculty of Technology, Jacob of Paradies University, 66-400 Gorzów Wielkopolski, Poland; 2 Faculty of Production Engineering and Materials Technology, Czestochowa University of Technology, 19 Armii Krajowej Av., 42-201 Czestochowa, Poland; 3 Department of Foundry and Welding, Rzeszow University of Technology, Powstańców Warszawy 12, 35-959 Rzeszow, Poland

**Keywords:** compacted graphite iron, austempering, austenitization, vermicular cast iron, AVGI

## Abstract

The significance of the matrix morphology of vermicular cast iron for the alloy’s thermal shock resistance was determined. The study included vermicular cast iron subjected to heat treatment in order to obtain an ausferritic matrix. Heat treatment involved austenitization at 960 °C for 90 min, followed by two different austempering variants at 290 °C and 390 °C, each for 90 min. Austempering at 390 °C resulted in a higher content of retained austenite compared to austempering at 290 °C. A test stand was used to determine thermal shock resistance, enabling repeated heating and cooling of the samples. The samples were heated inductively and subsequently cooled in water at a constant temperature of approximately 30 °C. The total length of cracks formed on the wedge-shaped surfaces of the tested samples was adopted as a characteristic value inversely proportional to the material’s thermal shock resistance. The samples heated to 500 °C were subjected to 2000 heating–cooling test cycles. It was found that in as-cast iron, structural changes were minor, whereas in the heat-treated material, changes in the structure were more noticeable. Under the influence of thermal shocks, ausferrite transforms into ferrite with carbides. Among the analyzed materials, the most resistant cast iron was the one austempered at 290 °C. Oxide precipitates were observed near cracks and graphite regions.

## 1. Introduction

The influence of austempering temperature on the microstructure and mechanical properties of compacted graphite iron or ductile iron is discussed by numerous researchers [1,2,3,4]. In study [5], the researchers analyzed the effect of heat treatment of compacted graphite iron on the morphology of graphite precipitates. They stated that, in the samples after heat treatment, a decrease in the degree of vermicularization of graphite is observed in comparison to the as-cast iron (the share of graphite precipitates with a shape close to spherical increases in the structure). The heat treatment also led to a reduction in the average size of graphite precipitates. Darmawan et al. [6] determined the effect of heat treatment, among others, on the microstructure of malleable cast iron. The applied heat treatment included austenitization at 850 °C for 60 min, followed by austempering at 300 °C for 30, 60, or 90 min. It was observed that longer holding times of 60 and 90 min resulted in an increase in the graphite particle size. Subjecting the cast iron to heat treatment through austenitization and austempering (within a specific range) results in the formation of an ausferritic structure, and an improvement in tensile strength, yield strength, and hardness, with a simultaneous decrease in material elongation [7,8,9]. The researchers in [10] analyzed the influence of heat treatment parameters—austenitization and austempering of compacted graphite iron—on the mechanical properties of the material. For this purpose, they used multi-criteria analysis, which allows for the assessment of correlations between treatment parameters and specific properties. A total of 27 heat treatments were performed with different parameters. The optimal conditions were assumed as maximum tensile strength and yield strength, and elongation of approximately 0.7%, and it was determined that the following heat treatment parameters: Tγ = 890 °C; Tpi = 290 °C; τγ = 120 min; τpi = 150 min, achieve these results. Akinribide et al. [11] analyzed unalloyed and alloyed ductile iron in terms of its microstructure, mechanical, and tribological properties. Austenitization was carried out within the temperature range of 900–970 °C for 60 min, followed by austempering at 350 °C for 30 min. It was observed, among other findings, that the alloyed ductile iron contains ferrite, austenite, and stabilized austenite, which contributed to the improvement of the material’s ductility.

Cast iron components operate under a variety of service conditions. Brake discs and drums, turbines, and exhaust manifolds, as well as castings used in the metallurgical or energy industries, often function under sudden temperature variations. Such thermal fluctuations can lead to rapid degradation of components; therefore, understanding damage mechanisms and implementing strategies for their prevention is essential. In study [12], vermicular cast iron subjected to high-frequency cyclic plasma and surface cooling airflow was investigated in order to better understand its behaviour and the underlying mechanisms under varying cooling conditions in a specific thermal shock environment. In another study [13], the authors sectioned samples from cylinder heads cast from two types of vermicular cast iron (with tensile strengths of 400 MPa and 450 MPa, respectively) and subjected them to thermal fatigue testing. The temperature ranges used in the tests were 25–420 °C, 25–450 °C, 25–550 °C, 25–650 °C, and 25–750 °C, with specimens containing pre-formed V-shaped notches. It was concluded that a higher pearlite content in the matrix leads to a lower thermal crack growth rate. Moreover, the temperature at which grain boundary sliding occurs in ferrite, as well as the transformation temperature of pearlite, play a significant role in the formation and propagation of thermal fatigue cracks in both types of vermicular graphite cast iron.

It is also worth noting that some researchers focus on the morphology of graphite precipitates, which also significantly influences crack propagation. For example, in the study [14], ductile and grey cast irons with ferritic matrices were investigated with respect to their potential use in casting moulds for the glass industry, as well as their resistance to thermal shock and thermal fatigue. The results indicated that graphite flakes present in grey cast iron behave as voids within the material, which increases susceptibility to brittle fracture and, consequently, reduces both impact strength and ductility. In contrast, ductile cast iron possesses approximately 40% higher resistance to thermal shock. In studies [15,16], two types of vermicular cast iron were compared in terms of their thermal fatigue resistance: one with a ferritic matrix (EN-GJV-350), and the other with a pearlitic matrix (EN-GJV-450). The tests were carried out under several temperature ranges, maintaining a constant T_min_ of 200 °C, while T_max_ varied from 620 °C to 750 °C. The criterion for thermal fatigue resistance was the number of thermal cycles leading to complete fracture of the specimen. The results showed that the ferritic matrix cast iron exhibited lower resistance to thermal fatigue. In the case of pearlitic vermicular cast iron, rapid heating and cooling led to the progressive transformation of the pearlitic matrix into ferrite. This ferritization process reduced the temporary strength and hardness of the material, ultimately resulting in specimen failure.

The aim of this study was to analyze the influence of austempering temperature on the thermal shock resistance of compacted graphite iron (CGI). To conduct the experiments, thermal shock simulations were performed using a custom-designed test stand. Thermal shock resistance, understood as the ability to withstand sudden temperature changes, was evaluated based on the length of surface cracks formed on the specimens. A similar approach was employed in study [17], where cast iron samples with different graphite morphologies were examined. The present work focused on assessing the effect of an ausferritic matrix obtained through austempering at two different temperatures on thermal shock performance. Sudden temperature fluctuations may lead to material cracking, reduced thermal conductivity, or high-temperature corrosion. Understanding these degradation mechanisms can support the development of more resistant materials and help implement strategies that improve the operational reliability of cast components.

## 2. Materials and Methods

The production process of the analyzed compacted graphite iron (CGI) was described in detail in study [5]. Table 1 presents the chemical composition of the produced CGI. Microstructural observations were carried out using an Olympus DSX1000 digital microscope (DSX1000 Software Ver. 1.2.5, Olympus, Shinjuku, Japan).

The CGI was subjected to heat treatment involving austempering. Based on preliminary studies [18], the literature data on the heat treatment of ductile iron reported by several authors [19,20,21,22,23], and studies on the heat treatment of CGI [24], the austenitization temperature (Tγ) was set at 960 °C. Austempering (Tpi) was carried out at two temperatures: 290 °C and 390 °C. The 100 °C temperature difference was intended to obtain structures with different proportions of ferrite and retained austenite. Both the austenitization time (τγ) and austempering time (τpi) were kept constant at 90 min. The parameters of the heat treatment process are summarized in Table 2.

**Figure 1:**
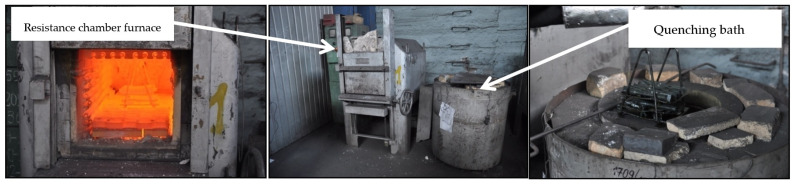
Workstation used for austenitization and austempering.

The heat treatment was carried out in industrial condition at “Odlewnie Polskie S.A.” in Starachowice, Poland (see Figure 1). Austenitization was performed in an electric resistance chamber furnace manufactured by ELTERMA (Świebodzin, Poland), in an air atmosphere. Austempering was conducted in a quenching bath, where a salt bath was used as the quenching medium. After removal from the bath, the specimens were rinsed to remove salt residues.

The microstructure of as-cast CGI was examined using samples taken from type IIb test castings according to standard PN-EN 16079:2012 [25]. The classification of graphite precipitates was performed in accordance with standard [26], while microstructural evaluation was conducted based on the methodology described in standard [27]. A similar procedure was applied to assess the microstructure after heat treatment. The amount of retained austenite was determined using the NIS-Elements D image analysis software (Version 4.5). Measurements were carried out at 72 locations under 400× magnification. Additionally, for heat-treated CGI samples, microstructural observations were performed using a Vega 3 scanning electron microscope (VEGA TC v3.6.0.0, Tescan, Brno, Czech Republic). Chemical composition analysis was also conducted using an Inca X-Act EDS system (Oxford Instruments, Abingdon, UK).

This study involved the evaluation of the tensile strength of CGI, both in the as-cast condition and after heat treatment. For this purpose, tensile specimens were machined from the test castings (type IIb) after separating them from the riser. To account for possible scale formation during thermal processing, the gauge diameter of the tensile specimens was intentionally increased from the typical 10 mm to 12 mm. The additional material was subsequently removed after the heat treatment. For each experimental variant, four tensile tests were carried out. The minimum sample size was determined based on statistical methods, assuming an allowable estimation error of 20 MPa. Mechanical testing was performed using a ZWICK 1488 universal testing machine (Ulm, Germany).

**Figure 2:**
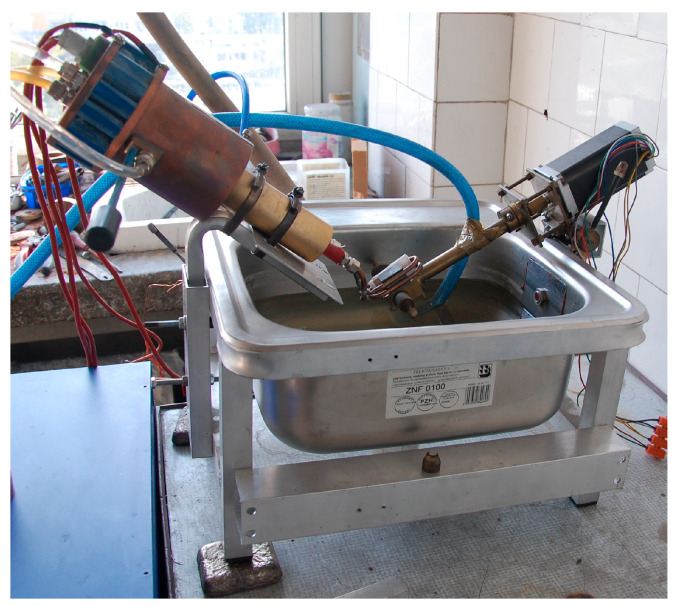
Experimental setup for testing materials under sudden temperature changes.

To evaluate the thermal shock resistance of CGI, a dedicated device was used, designed, and constructed at the Czestochowa University of Technology. The system enables repeated heating and cooling of test specimens within a controlled temperature range. A detailed description of the apparatus and testing methodology is provided in studies [14,28,29]. Figure 2 presents the test apparatus used in this study.

The equipment is equipped with a high-frequency induction heater (operating at 100 kHz). The temperature of the heated specimen was monitored using a stationary Marathon 2M pyrometer (Raytek, Santa Cruz, CA, USA). After reaching the target temperature (measured via pyrometry), the specimen was automatically rotated 180°, immersing the heated part into a water tank, while simultaneously initiating the heating of the opposite end. It should be noted that the water temperature of approx. 30 °C was maintained at a constant level using a heat exchanger system. The samples were heated for approx. 14 s and then cooled in water for the same duration.

**Figure 3:**
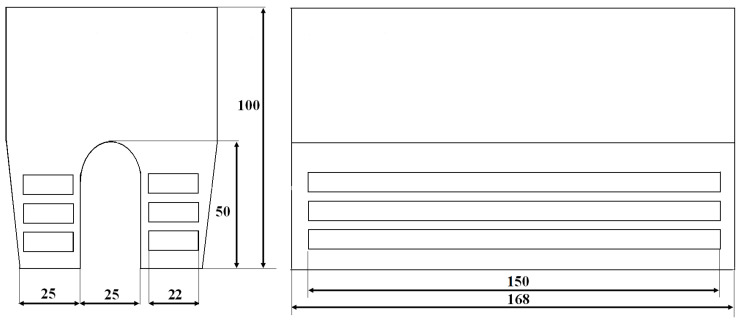
Location of specimen extraction for thermal shock testing from type IIb casting.

**Figure 4:**
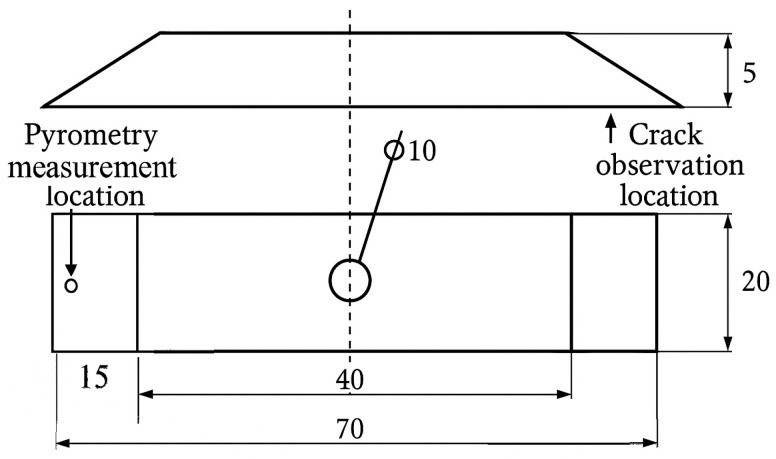
Specimen used for thermal shock resistance evaluation.

For the thermal shock resistance tests, flat specimens with a length of 70 mm and a thickness of 5 mm were used. Both ends of the specimens were tapered over a length of 15 mm. The samples made of CGI were cut from type IIb test castings (see Figure 3). The geometry and dimensions of the specimens used for thermal shock testing—both in the as-cast condition and after heat treatment (after machining off the surface layer)—are shown in Figure 4. This figure also indicates the temperature measurement point (for pyrometry), as well as the crack observation areas (both tapered ends of the specimen).

The specimens were subjected to the following sequence of steps:

– Heating of the wedge-shaped end using an induction coil until the target temperature was reached;
– Rotating the specimen by 180°;
– Cooling in water during the heating of the opposite end.

The total length of all microcracks observed on the wedge-shaped surfaces of each specimen was adopted as the measure of thermal shock resistance. Preliminary crack length assessment was performed using a non-destructive technique—penetrant testing. Final measurements and observations of crack length were carried out using an Olympus DSX1000 digital microscope. The distribution of elements in vermicular cast iron in the presence of cracks was investigated using an Energy Dispersive Spectroscopy (EDS) detector integrated into an Axia ChemisSEM scanning electron microscope (SEM) from ThermoFisher Scientific (Waltham, MA, USA). The microscope is equipped with a tungsten filament electron source. The analyses were performed at magnifications of 6000× and 25,000× using an accelerating voltage of 30 kV.

## 3. Results and Discussion

### 3.1. Microstructure Analysis

**Figure 5:**
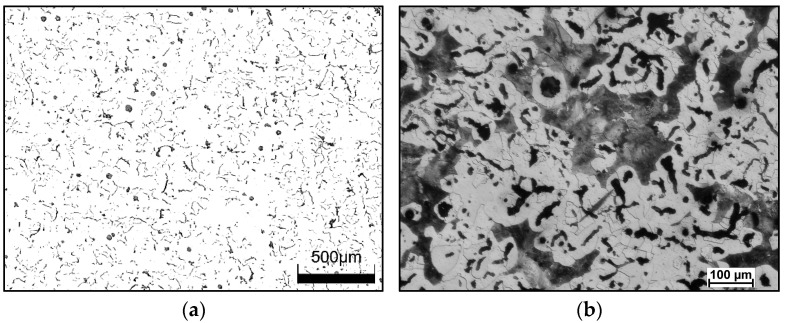
Cast iron with vermicular graphite as-cast state; (**a**) shape and size of graphite particles, nonetched specimen; (**b**) microstructure of cast iron, metallographic specimen etched with Nital.

Figure 5 shows the graphite morphology and the microstructure of CGI cast iron. In the examined cast iron, vermicular graphite accounted for approximately 95%, while nodular graphite made up a small portion of about 5%. The ferrite content slightly exceeded 50%, while the remainder of the matrix consisted of pearlite.

**Figure 6:**
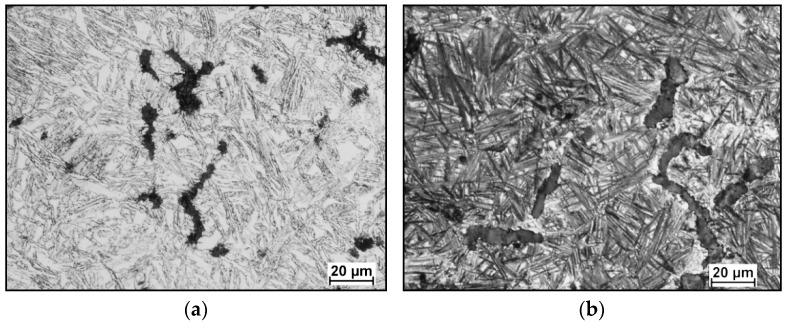
Microstructures of CGI after heat treatment. Heat treatment parameters: (**a**) Tγ = 960 °C, Tpi = 390 °C; (**b**) Tγ = 960 °C, Tpi = 290 °C; etched with nital, magnification 500×.

Microstructural analysis of CGI subjected to austempering revealed that the matrix consisted of so-called ausferrite, i.e., a structure composed of acicular ferrite and retained austenite (see Figure 6). To determine the amount of retained austenite, the matrix was analyzed using the NIS-Elements D image analyzer. The measurements were performed at 72 points under 400× magnification. The results showed that the highest retained austenite content (approx. 38%) was observed in samples austempered at 390 °C. Samples subjected to austempering in a salt bath at 290 °C exhibited a lower retained austenite content of approx. 24%

**Figure 7:**
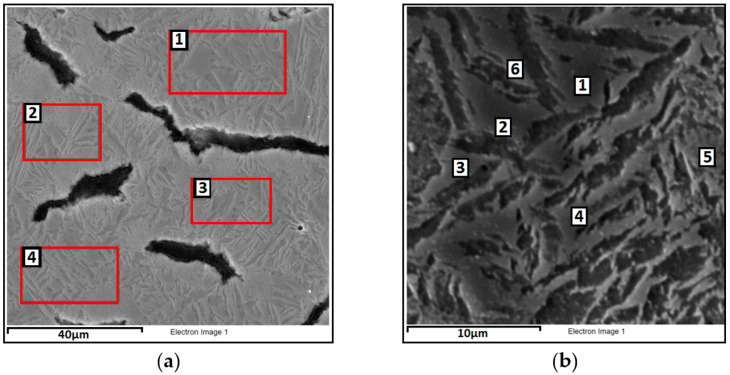
SEM image of compacted graphite cast iron after heat treatment, austempered at 390 °C, showing the locations used for quantitative chemical composition analysis: (**a**) surface analysis (magnification 2000×); (**b**) point analysis (magnification 8000×).

**Figure 8:**
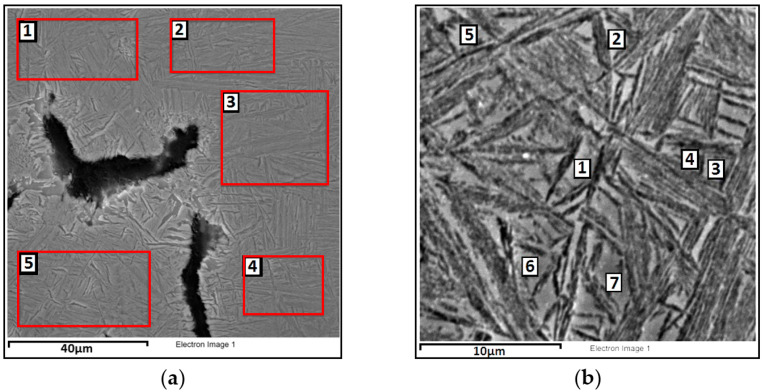
SEM image of compacted graphite cast iron after heat treatment, austempered at 290 °C, showing the locations used for quantitative chemical composition analysis: (**a**) surface analysis (magnification 2000×); (**b**) point analysis (magnification 8000×).

For heat-treated CGI, microstructural observations were performed using a scanning electron microscope, along with chemical composition analysis. At magnifications of 2000× and 8000×, chemical composition was analyzed on selected surface areas and specific points to identify differences resulting from the heat treatment process. The microstructure is shown in Figure 7 and Figure 8, and the results of the observations are summarized in Table 3 The analysis includes elements such as C, Si, Fe, and Cu, as their content in the alloy exceeds 1 wt%. Other elements are present only in trace amounts, and were therefore not reported.

Analyzing the alloy subjected to heat treatment at an austempering temperature of 390 °C, it can be stated that retained austenite exhibits a higher average content of carbon (C = 3.77%) and copper (Cu = 1.06%) compared to ausferrite, with a simultaneously lower iron content. The silicon level is similar in both phases. Analyzing variant 2, i.e., the alloy austempered at a lower temperature of 290 °C, an increase in carbon content is observed in both ausferrite (C = 5.61%) and retained austenite (C = 4.33%) compared to variant 1. The copper content in variant 2 is higher in ausferrite (Cu = 1.04%) than in austenite (Cu = 0.71%). The content of both carbon and copper in ausferrite and retained austenite clearly depends on the applied heat treatment parameters. As confirmed by studies [30,31], lower austempering temperatures promote carbon enrichment in retained austenite, which enhances the mechanical strength and thermal stability of the material.

### 3.2. Mechanical Properties

Table 4 presents the average values of basic mechanical properties—tensile strength (R_m_), yield strength (R_p_), elongation (A_5_), and Brinell hardness (HB)—for compacted graphite cast iron in both the as-cast and heat-treated conditions. Tensile tests were conducted on four specimens per condition, while hardness measurements were performed twelve times for each variant. The hardness of the material was tested using a Brinell hardness tester, with a 2.5 mm diameter ball and a load of 1839 N. Mechanical strength tests were performed in accordance with the standard [32], and the hardness of the material was determined in accordance with the standard [33]. The highest tensile strength, yield strength, and hardness, accompanied by low elongation, were observed in the cast iron austempered at 290 °C.

### 3.3. Thermal Shock Resistance

**Figure 9:**
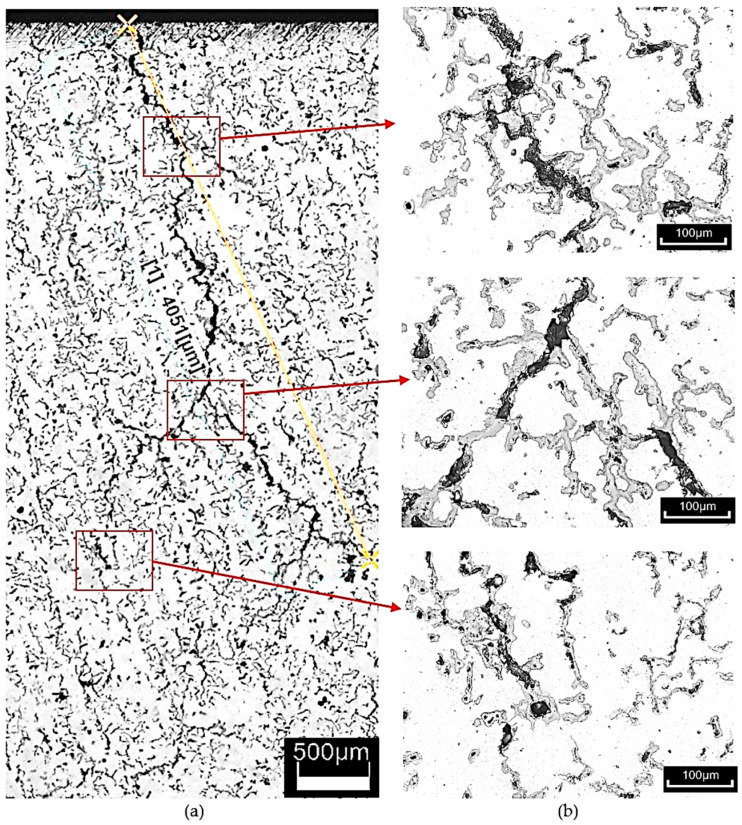
Example of cracks observed in compacted graphite cast iron after heat treatment (**a**) composite image from photos taken at 123× magnification, total area: 2582 × 5428 µm; (**b**) magnification 610×, unetched sample. The yellow line indicates the crack measurement.

The thermal shock resistance of cast iron was tested by heating the specimens to 500 °C, followed by cooling in water at approximately 30 °C. A total of 2000 heating and cooling cycles were performed. Upon observing the cracks, it was noted that they propagated along the grains of vermicular graphite. In this material, crack branching occurred, forming a so-called crack network. The crack length was measured from the edge of the specimen to the point where the crack disappeared. An example of a crack is shown in Figure 9. The total crack length on the wedge-shaped sections was as follows:

- In the as-cast condition—25,676 µm;
- After heat treatment at Tpi = 390 °C (Variant 1)—21,740 µm;
- After heat treatment at Tpi = 290 °C (Variant 2)—15,628 µm.

**Figure 10:**
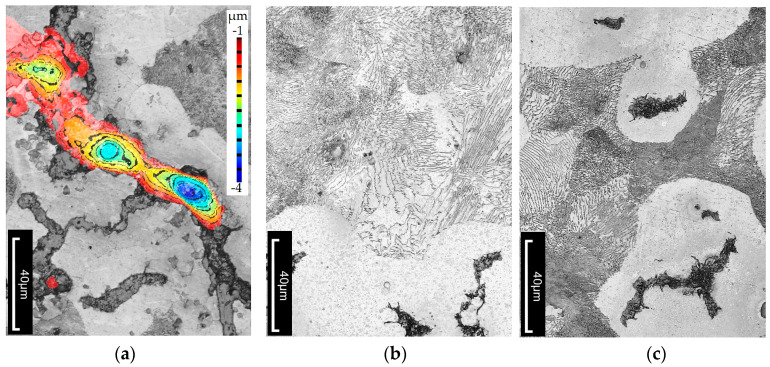
Microstructure of as-cast compacted graphite cast iron. The alloy was heated to 500 °C over 2000 thermal cycles. Etched with nital. (**a**) Example of a crack with an indicated depth. (**b**,**c**) Microstructure.

**Figure 11:**
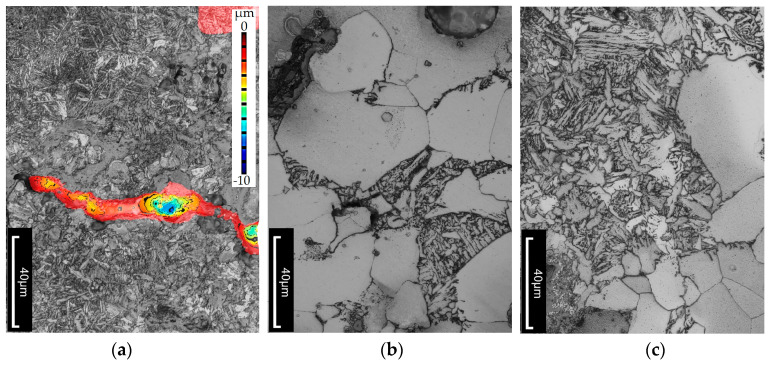
Microstructure of compacted graphite cast iron austempered at Tpi = 390 °C. The alloy was heated to 500 °C over 2000 thermal cycles and exhibited the greatest total crack length. Etched with nital. (**a**) Example of a crack with an indicated depth. (**b**,**c**) Microstructure.

**Figure 12:**
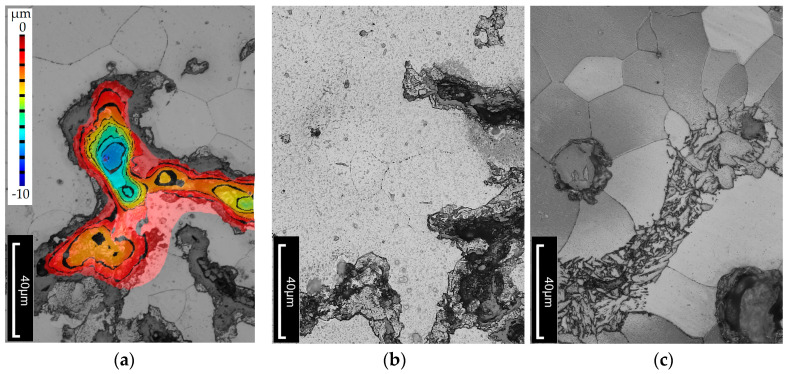
Microstructure of compacted graphite cast iron austempered at Tpi = 290 °C. The alloy was heated to 500 °C over 2000 thermal cycles. Etched with nital. (**a**) Example of a crack with an indicated depth. (**b**,**c**) Microstructure.

An additional objective of the study was to determine whether, and to what extent, subjecting the cast iron to thermal shock leads to changes in the microstructure of the material. Metallographic cross-sections were prepared from the wedge-shaped ends of the tested specimens. Microstructures were analyzed at various distances from the specimen edge. Images were also taken near the cutting edge of the specimen, where cracks were visible. The topography of the crack surfaces was mapped using the LEXT software (DSX1000 Software Ver. 1.2.5) integrated with the Olympus DSX1000 digital microscope. Examples of the observed structural changes are shown in Figure 10, Figure 11 and Figure 12. It should be emphasized that the temperature distribution within the wedge-shaped section of the specimen was not uniform. The outer edge of this section was subjected to the highest thermal load, with the degree of heating decreasing with distance from the edge. Therefore, microstructural observations were carried out at different distances from the heated area.

It should be noted that under the influence of varying temperatures reaching up to 500 °C, as well as thermal stresses during testing, slight structural changes are visible in as-cast vermicular graphite cast iron. Figure 10 shows the microstructure of vermicular graphite cast iron after thermal shock. The figure includes an example of a crack with an indicated depth (see Figure 10a). It can be seen that the microstructure consists of graphite, ferrite, and pearlite. In the ferritic–pearlitic matrix, as the temperature increases, the pearlite (Fe_3_C + ferrite)—specifically the cementite—begins to decompose, releasing carbon. This carbon diffuses toward regions with lower chemical potential, such as prior austenite grain boundaries or interphase boundaries. The described mechanism was confirmed by researchers in study [34,35,36], who observed similar microstructural behaviour under thermal exposure.

In the case of heat-treated cast iron, microstructural changes can be observed. Retained austenite, which is metastable at both room and elevated temperatures, tends to undergo phase transformation [37,38,39,40]. A higher amount of retained austenite reduces thermal conductivity, which leads to thermal stresses and cracking—as was the case for the specimen austempered at 390 °C, where the retained austenite content reached 38%. It can be concluded that a high proportion of austenite makes the material less resistant to elevated temperatures. In this specimen, structural changes occur more slowly than in the one austempered at 290 °C. In the microstructure (see Figure 11), carbide precipitates—especially cementite—are visible, mainly along phase and grain boundaries, as well as within ferrite. Fine ferrite needles begin to coalesce, causing the structure to thicken. In this case, ferrite occupies approximately 20% of the matrix, while the rest remains untransformed. In contrast, the specimen austempered at 290 °C exhibits a predominantly ferritic structure (about 90%) with 10% of the matrix still retaining untransformed structure.

**Figure 13:**
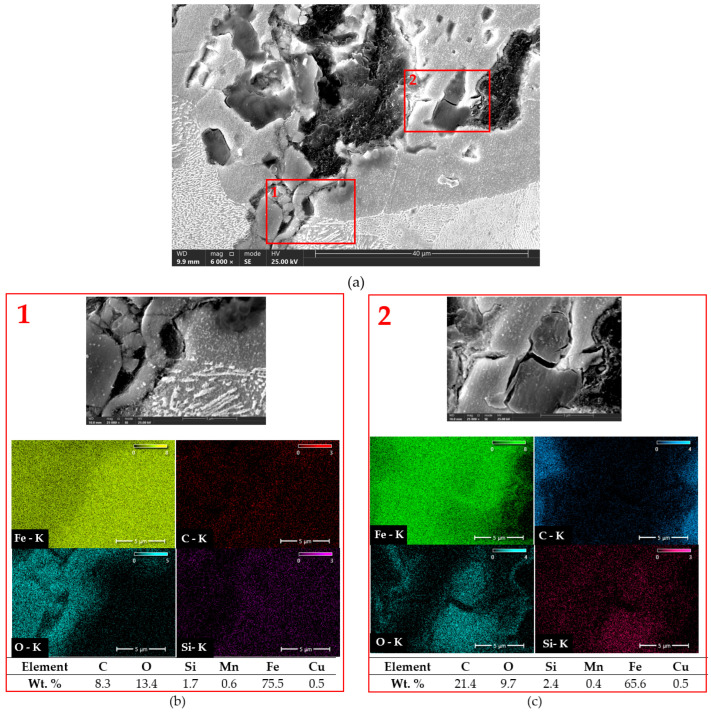
Analysis of elemental distribution in as-cast compacted graphite cast iron. (**a**) Microstructure at 6000× magnification. Red rectangles indicate the locations of chemical composition measurements. (**b**,**c**) Selected areas from (**a**) shown at 25,000× magnification with EDS analysis.

**Figure 14:**
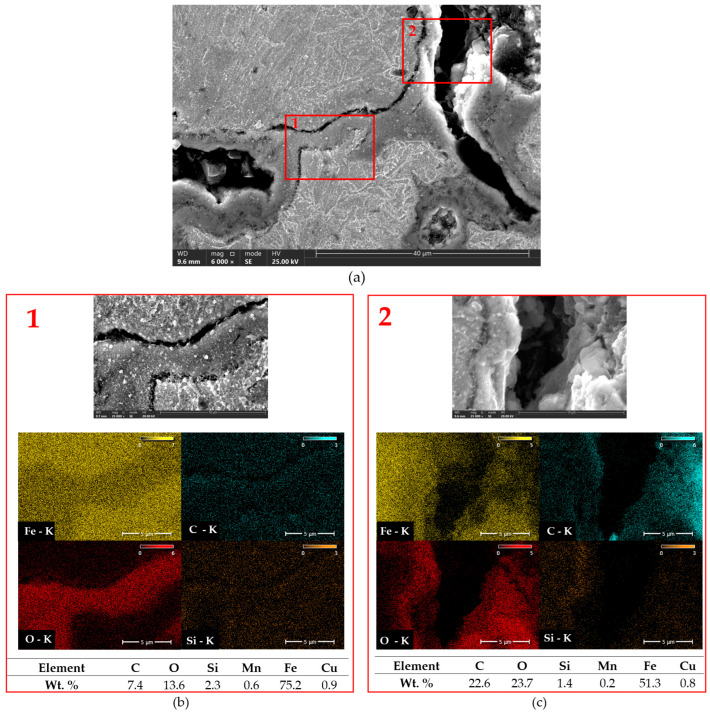
Analysis of area distribution of elements in compacted graphite cast iron austempered at Tpi = 390 °C. (**a**) Microstructure at 6000× magnification. Red rectangles indicate the locations of chemical composition measurements. (**b**,**c**) Selected areas from (**a**) shown at 25,000× magnification with EDS analysis.

**Figure 15:**
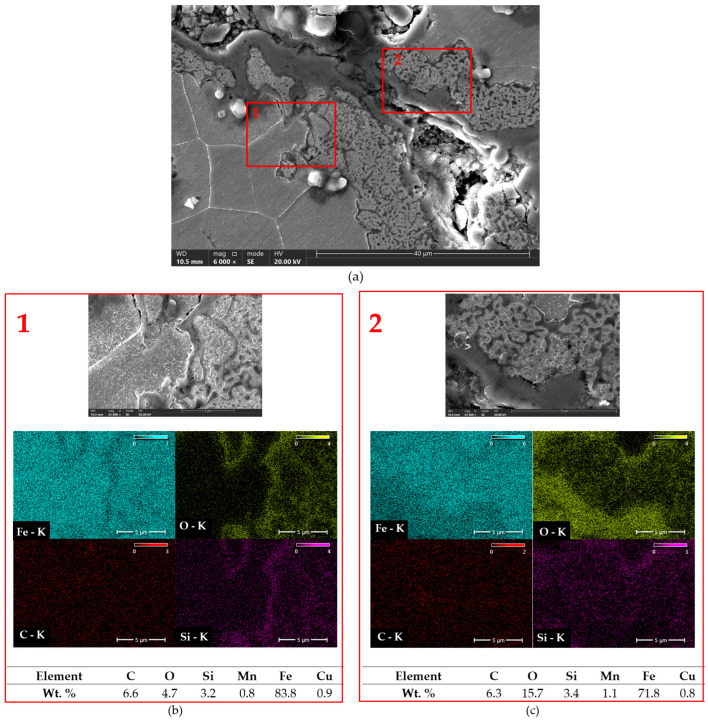
Analysis of area distribution of elements in compacted graphite cast iron austempered at Tpi = 290 °C. (**a**) Microstructure at 6000× magnification. Red rectangles indicate the locations of chemical composition measurements. (**b**,**c**) Selected areas from (**a**) shown at 25,000× magnification with EDS analysis.

Figure 13, Figure 14 and Figure 15 show example cracks in the analyzed compacted graphite cast iron specimens after thermal shock, along with point EDS analysis. Prior to thermal shock exposure (see Figure 6), no oxides were observed in the vicinity of vermicular graphite. After testing, oxidation-affected zones can be seen. Oxides were detected within clusters of vermicular graphite and near cracks. Oxygen readily penetrates the matrix and diffuses through it, leading to the formation of oxidation products surrounding the graphite. Figure 13 presents the microstructure with a representative crack in compacted graphite cast iron with a ferritic–pearlitic matrix. The EDS results confirm the presence of oxygen, especially in the crack regions, indicating significant surface oxidation of the material. It may be assumed that small concentrations of silicon could suggest the possible formation of silicon oxides. In this type of cast iron, however, the effects of high-temperature corrosion appear to be minimal.

Figure 14 and Figure 15 present SEM images with EDX analysis of compacted graphite cast iron subjected to austempering. It is clearly visible that the cracks caused by thermal shock propagated along phase boundaries, and that oxidation occurred intensively both along the cracks and at phase interfaces. The rapid temperature changes induced thermal stresses that led to crack propagation, while the simultaneous presence of water/air and elevated temperatures favoured the formation of iron oxides (Fe_2_O_3_, Fe_3_O_4_), as well as silicon oxides (SiO_2_). Figure 14 shows austempered compacted graphite cast iron (Tpi = 390 °C) with a clearly visible crack. Distinct crack branching is observed, mainly following intergranular paths. Based on EDX analysis, a high carbon concentration was detected in areas corresponding to the location of graphite, as well as within the crack itself, which may indicate the presence of graphite degradation products. In Figure 15a, the microstructure of a prior austenite grain boundary with an associated crack is visible. Figure 15b,c, taken at 25,000× magnification, show clear intergranular crack zones with features characteristic of brittle fracture. In these regions, EDX analysis revealed increased oxygen concentration, indicating oxidation processes typical of high-temperature corrosion induced by thermal shocks. The distribution of silicon suggests the possible formation of silicon oxides [41,42,43].

The analysis clearly suggests that the oxidation process occurred intensively along the cracks and at the phase boundaries. With repeated thermal shocks, the resulting thermal stresses led to crack propagation, and the access of air and high temperature promoted the formation of iron oxides (Fe_2_O_3_, Fe_3_O_4_) and, most likely, silicon oxides (SiO_2_), if there was a silicon admixture in the composition.

## 4. Conclusions

Based on the conducted studies on compacted graphite iron in relation to the thermal shock resistance of this material, the following conclusions can be drawn:

1. Samples subjected to austempering at 390 °C exhibited a higher content of retained austenite (approximately 38%) compared to samples austempered at 290 °C (approximately 24%).
2. Samples austempered at 290 °C are characterized by a higher carbon content in the ausferrite compared to the samples from variant no. 1 (austempered at 390 °C), which may be the reason for higher thermal stability and mechanical strength of this microstructure.
3. The applied heat treatment of vermicular cast iron resulted in an increase in tensile strength, yield strength, and hardness, accompanied by a decrease in elongation. Samples austempered at 290 °C exhibited the most significant changes in mechanical properties compared to the as-cast condition. It should be emphasized that these samples also demonstrated the highest resistance to thermal shocks.
4. After conducting the experiment and analyzing the effect of thermal shocks on microstructural changes, it can be stated that in vermicular cast iron in the as-cast state, no significant structural changes were observed. In the case of CGI, a transformation of the matrix from ausferritic to a ferritic matrix with carbides was noted. In the sample austempered at 390 °C, ferrite occupies approximately 20% of the matrix, while the remaining part is composed of the untransformed ausferritic matrix. In the sample austempered at 290 °C, the matrix is composed of approximately 90% ferrite and 10% untransformed microstructure.
5. It can be concluded that a high austenite content reduces the material’s resistance to rapid and variable temperature fluctuations.
6. Thermal shock exposure resulted in surface oxidation of the material, particularly along cracks and graphite regions. This led to increased high-temperature corrosion of the compacted graphite iron after austempering, compared to the as-cast condition.

## Figures and Tables

**Table 1:** Chemical composition (wt.%) of vermicular cast iron.

C	Si	Mn	Cu	P	S	Mg
3.22	2.38	0.192	1.02	0.054	0.022	0.0271

**Table 2:** Heat treatment parameters of CGI.

Designation of Heat Treatment Variants	Austenitization Temperature Tγ [°C]	Austenitization Time τγ [min]	Austempering Temperature Tpi [°C]	Austempering Time τpi [min]
**Variant 1**	960	90	390	90
**Variant 2**	960		290	

**Table 3:** Results of quantitative chemical composition analysis in ausferrite and retained austenite of compacted graphite cast iron after heat treatment, for the areas and points marked in Figure 7 and Figure 8.

Heat Treatment Variant of Cast Iron and Analyzed Structural Constituents			Fraction (%)			
			C, %	Si, %	Fe, %	Cu, %
Variant 1	Content in ausferrite—Figure 7a	1	2.98	2.85	93.32	0.85
		2	3.75	2.58	92.85	0.82
		3	2.78	2.61	93.84	0.77
		4	2.61	2.65	93.90	0.84
	Average:		**3.03**	**2.67**	**93.48**	**0.82**
	Content in austenite—Figure 7b	1	3.87	2.92	92.22	0.99
		2	3.65	2.81	92.46	1.08
		3	3.56	2.88	92.33	1.22
		4	3.63	2.94	92.27	1.16
		5	4.64	2.84	91.58	0.94
		6	3.29	2.89	92.85	0.97
	Average:		**3.77**	**2.88**	**92.29**	**1.06**
Variant 2	Content in ausferrite—Figure 8a	1	6.07	2.75	90.11	1.07
		2	6.08	2.61	90.25	1.06
		3	4.85	2.82	91.25	1.07
		4	4.96	2.73	91.28	1.03
		5	6.07	2.62	90.34	0.97
	Average:		**5.61**	**2.71**	**90.65**	**1.04**
	Content in austenite—Figure 8b	1	4.49	2.46	92.32	0.73
		2	4.15	2.73	92.17	0.96
		3	4.54	2.52	91.89	0.79
		4	5.04	2.55	91.39	0.78
		5	4.61	2.66	92.07	0.66
		6	3.69	2.46	93.10	0.56
		7	3.80	2.23	93.20	0.51
	Average:		**4.33**	**2.52**	**92.31**	**0.71**

**Table 4:** Tensile strength (R_m_), elongation (A_5_), yield strength (R_p_), and Brinell hardness (HB) values for compacted graphite cast iron in the as-cast and heat-treated conditions.

Tensile Strength R_m_ [MPa]	Yield Strength R_p_ [MPa]	Elongation A_5_ [%]	Hardness HB [MPa]
Compacted graphite cast iron (as-cast)			
371	331	2.1	183
Austempered compacted graphite cast iron at 390 °C			
623	485	1.6	260
Austempered compacted graphite cast iron at 290 °C			
824	584	0.7	334

## Data Availability

The original contributions presented in this study are included in the article. Further inquiries can be directed to the corresponding author.
